# Drift Reduction of a 4-DOF Measurement System Caused by Unstable Air Refractive Index

**DOI:** 10.3390/s20216329

**Published:** 2020-11-06

**Authors:** Ruijun Li, Yongjun Wang, Pan Tao, Rongjun Cheng, Zhenying Cheng, Yongqing Wei, Xueming Dang

**Affiliations:** School of Instrument Science and Opto-electronics Engineering, Hefei University of Technology, Hefei 230009, China; rj-li@hfut.edu.cn (R.L.); wangyongjun@mail.hfut.edu.cn (Y.W.); 2017170020@mail.hfut.edu.cn (P.T.); chengzhenying01@hfut.edu.cn (Z.C.); yqwei_hfut@hfut.edu.cn (Y.W.); dangxm@hfut.edu.cn (X.D.)

**Keywords:** laser measurement system, beam drift, bellows, fluid analysis

## Abstract

Laser beam drift greatly influences the accuracy of a four degrees of freedom (4-DOF) measurement system during the detection of machine tool errors, especially for long-distance measurement. A novel method was proposed using bellows to serve as a laser beam shield and air pumps to stabilize the refractive index of air. The inner diameter of the bellows and the control mode of the pumps were optimized through theoretical analysis and simulation. An experimental setup was established to verify the feasibility of the method under the temperature interference condition. The results indicated that the position stability of the laser beam spot can be improved by more than 79% under the action of pumping and inflating. The proposed scheme provides a cost-effective method to reduce the laser beam drift, which can be applied to improve the detection accuracy of a 4-DOF measurement system.

## 1. Introduction

Among various non-contact measurement systems, laser measurement systems are widely used in the detection of machine tool errors because of their high accuracy and efficiency, such as the position and angular errors of large gantry machine tools. A four degrees of freedom (4-DOF) measurement system is currently the most widely used method for the merits of measuring position and angle errors at the same time. However, the laser beam drift caused by the unstable air refractive index greatly decreases the detection accuracy, especially at long distances, thus propagating the errors. With the continuous improvement of the machining accuracy of machine tools, the requirements for detection accuracy have increased. Therefore, reducing the laser beam drift to improve the detection accuracy of laser measurement systems is an urgent concern.

During the past decade, a series of methods have been developed to reduce the influence of the laser beam drift on measurement results, such as the double beam collimation, common path compensations, feedback compensations, dual wavelength methods, and refractometer methods. The preceding three methods are used to suppress laser beam drift by improving the optical path and structure. The double beam collimation method adopts a special design of optical path structure to suppress the beam drift. For example, Zhao et al. proposed a new kind of displacement and angle interferometer with only one reference corner cube retroreflector [1]. Chen et al. presented a novel straightness and coaxiality measurement system [2]. The double beam collimation method is slightly affected by atmospheric disturbances and can effectively suppress the beam drift. With this method, the laser beam stability can be improved by 50% when the measuring distance is 300 mm. However, many components should be used, making the method inconvenient for adjustment. In the common path compensation method, beam drift is detected and compensated according to the measurement results. For example, Chen et al. proposed a heterodyne straightness and displacement measuring interferometer for laser beam drift compensation [3,4,5]. Feng et al. and Huang et al. arranged a beam splitter in front of the measuring retroreflector to detect the beam drift and thus compensate for the small angular displacement. Through this method, the measurement error is within 0.8 μm when the measurement distance is 500 mm [6,7,8]. The angular drift in the straightness error can be compensated using this method, especially when the drift is caused by the laser itself. However, the compensation effect is not significant for other factors, such as atmospheric disturbances. The feedback compensation method is introduced on the basis of the common path compensation method. The angular and horizontal drifts of the beam are monitored separately, and the measurement results are compensated in real time. Zhu et al. placed a semi-reflective film on the right-angle prism to detect and compensate for the beam drift [9,10,11]. Huang et al. and Zhao et al. employed a PZT-actuated turning a mirror to compensate for the beam drift in real time. Through this method, the offset angles can be compensated to ± 0.01 arc sec during 1.5 h when the measurement distance is 450 mm [12,13]. The drift error caused by multiple factors can be corrected using feedback compensation method to improve the control accuracy of the beam drift. However, a control system with high stability and precision is required, which results in high cost. Compensation is commonly used for the above methods, which do not focus on the cause of the laser beam drift. So far, they are not used for long distance measurement system.

In fact, the change of air refractive index is believed to be the main reason for laser beam drift. Then, the dual wavelength methods and refractometer methods are proposed successively, and the laser beam drift is reduced by measuring the refractive index of air. Two different wavelength lasers are utilized to calculate for the refractive index gradient of air and correct measurement results for the dual wavelength method. Meiners-Hagen et al. and Matsumoto et al. developed a two-color interferometer by using lasers with different wavelengths as light sources to measure the variation of air refractive index for length measurement [14,15,16]. This method can be applied to correct the laser beam drift in long-distance measurement over several kilometers, but the optical path structure of the system is complicated, and the cost is high. The refractometer methods utilize a refractometer to measure the refractive index gradient of air, then the measurement results can be corrected accordingly. Naoi et al. combined a ranging interferometer and a refractometer into an instrument, which can directly compensate for the influence of air refractive index in the measurement process [17,18,19]. However, it cannot accurately measure the refractive index on the axis of laser beam. To settle this problem, Lazar. J. proposed a dual counter measurement interferometer that combines a tracking refractometer with a displacement interferometer [20,21]. The accurate air refractive index can be obtained by this method. However, it cannot be applied to the 4-DOF measurement system that is without interference optical path.

In this study, a cost-effective and simple method by stabilizing the air refractive index is presented to reduce the laser beam drift. Compared with the existing methods, it restrains the generation of drift in principle from the influence mechanism in the laser propagation process. A bellows was utilized as a laser beam shield, and air pumps were employed to control the temperature and pressure in the bellows to create a stable propagation environment for the laser optical path.

## 2. Principle and Analysis

### 2.1. The Principle of the 4-DOF Measurement System

**Figure 1:**
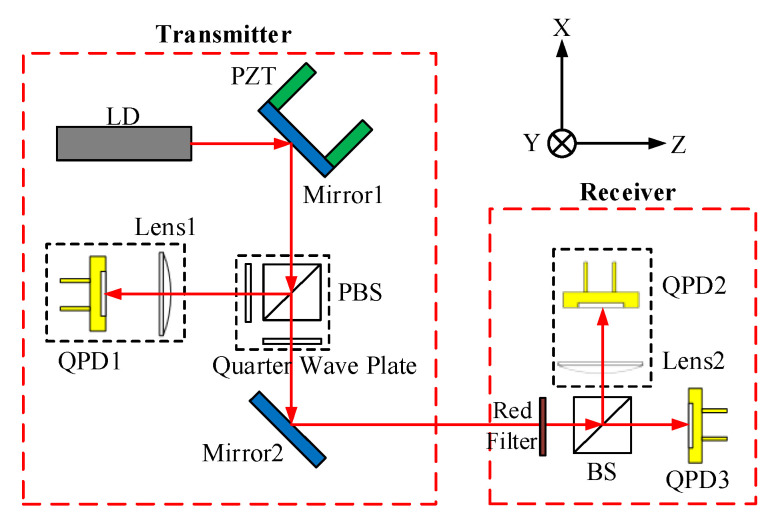
Principle of the four degrees of freedom (4-DOF) measurement system.

Generally, the 4-DOF measurement system is composed of a transmitter and a receiver (Figure 1) [22]. In order to measure the position and angle errors of machine tools, the transmitter is fixed on the stationary front end of a guide rail and the receiver is fixed on the moveable plate of the guide rail. The parallel beam emitted by the laser diode (LD) at the transmitter is divided into two vertical linear polarized beams (S-polarized light and P-polarized light) by the polarizing beam splitter (PBS). The S-polarized light is converged to the quadrant photodiode 1(QPD1) of the lens focal plane after passing through a quarter wave plate, then the angle drift error of the beam can be compensated. The polarization direction of p-polarized light changes by 45° compared with the original direction after passing through the 1/4 wave plate, so the output power of the LD can be ensured for the reason that the beam cannot be reflected back. The p-polarized light is filtered by a red-light filter after entering the receiver, and then it is divided into two non-linear polarized beams by the beam splitting prism (BS). One beam converges to the QPD2 on the focal plane of the lens, which is used as the reference for detecting the angle error of the machine tool guide rail. The other beam is directly incident on the QPD3 as the reference for detecting the straightness error of the machine tool guide rail.

### 2.2. Influence Mechanism of Laser Beam Drift

The laser beam travels in a straight path in a vacuum or isotropic medium. However, the actual atmospheric environment can hardly satisfy these conditions. In this case, refraction occurs when the laser beam passes through the interface of the different refractive index, thus resulting in laser beam drift.

Under standard environmental conditions, the refractive index of air can be calculated using the Ciddor formula as follows [23]:

(1) $$\begin{array}{l} N=(\frac{273.15}{1013.25}\cdot \frac{p}{T}\cdot N_{sph})-11.27\frac{e}{T}\\ N_{sph}=287.6155+\frac{1.62887}{\lambda^{2}}+\frac{0.01360}{\lambda^{4}} \end{array},$$

where *T* is the air temperature, *P* is the pressure, *e* is the vapor pressure, *N* is the refractive index, and *N_sph_* is the atmospheric phase refractivity.

The partial derivative of Equation (1) with respect to the parameters of temperature, pressure, and relative humidity can then be obtained as follows:

(2) $$\frac{dn}{dT}=-1.0967,\;\frac{dn}{dP}=0.295645,\;\frac{dn}{de}=-0.0413,$$

Equation (2) reveals that vapor pressure changes have the least effect on the refractive index among the three parameters, making it negligible. Therefore, only air temperature and pressure are the key influencing factors.

**Figure 2:**
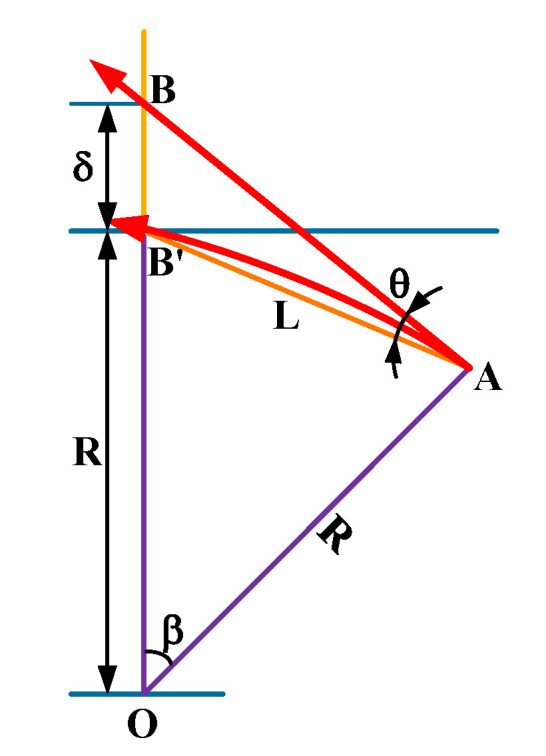
Computational model of beam drift.

A computational model is established to evaluate the drift of the laser beam (Figure 2). Assuming that the air refractive index in the bellows is uniformly distributed in the radial direction, the laser beam deviates from a straight line during propagation because of the continuous change of the air refractive index in the axial direction. Figure 2A,B represent the start and end points of the laser beam during measurement, respectively. The arc AB′ is used to approximately represent the actual trajectory of the optical path.

Next, Equation (3) is derived from the curvature of an arc, the curvature radius *R* of the actual trajectory AB′ of the optical path can be obtained as follows:

(3) $$R=\frac{1}{dn/dl},$$

where *dn/dl* is the axial gradient of the air refractive index.

On the basis of advanced mathematics and sine theorem, the angular variation *θ* of optical path can be approximated as

(4) $$\theta \approx \sin\theta =\sin\frac{\beta }{2}=\frac{L}{2R}=\frac{L}{2}\frac{dn}{dl},$$

where *L* is the optical path length. 

Similarly, the drift can be approximately derived as

(5) $$\delta \approx L\theta =\frac{L^{2}}{2}\frac{dn}{dl},$$

When the influence of relative humidity is ignored on Equations (2) and (5), the laser beam drift can be given as follows:

(6) $$\begin{array}{l} \delta =\frac{L^{2}}{2}\frac{dn}{dl}=\frac{L^{2}}{2}\left(\frac{dn}{dt}\times \frac{dt}{dl}+\frac{dn}{dp}\times \frac{dp}{dl}\right)\\ \;\;=\frac{L^{2}}{2}\left(-1.0967\times \frac{dt}{dl}+0.295645\times \frac{dp}{dl}\right) \end{array},$$

where *dt/dl* and *dp/dl* are the axial temperature and pressure gradient, respectively.

Equation (6) indicates that the laser beam drift increases linearly with the increase of axial temperature and pressure gradients. Thus, the stability of temperature and pressure should be improved to enhance the stability of the laser beam.

### 2.3. Maximum Allowable Axial Temperature and Pressure Gradient

Generally, high machining accuracy is required for precision machine tools, and the typical straightness error of the linear guide is less than 2 μm/m, such as the precision gantry grinding machine manufactured by FAVRETTO in Italy and the ultra-precision gantry grinder machine manufactured by Sumitomo in Japan. Supposing that the effective stroke of the linear guide of a high-precision machine tool is 2 m, the straightness error should be less than 4 μm. Accordingly, this error can be defined as 4 μm. The additional error caused by laser beam drift will be contained in the measurement result when this straightness error is measured by a laser measurement system. When the additional error is less than 1/5 of the straightness error (0.8 μm), it can be used to measure this straightness error according to the 3σ criterion. Based on Equation (6), when the temperature change in the bellows is only considered, the maximum axial temperature gradient can be approximately calculated as follows:

(7) $${\left(\frac{dt}{dl}\right)}_{\max}=\frac{2.6911\times 10^{5}\delta }{L^{2}}\approx 0.4{\;}^{{}^{\circ}}C/m,$$

Similarly, the maximum axial pressure gradient can be approximately calculated as

(8) $${\left(\frac{dp}{dl}\right)}_{\max}=\frac{9.3284\times 10^{7}\delta }{L^{2}}\approx 138\;\mathrm{Pa}/m,$$

Hence, the maximum allowable axial temperature and pressure gradients are 0.4 °C/m and 138 Pa/m, respectively.

## 3. Scheme Design and Parameter Optimization

### 3.1. Scheme Design

**Figure 3:**
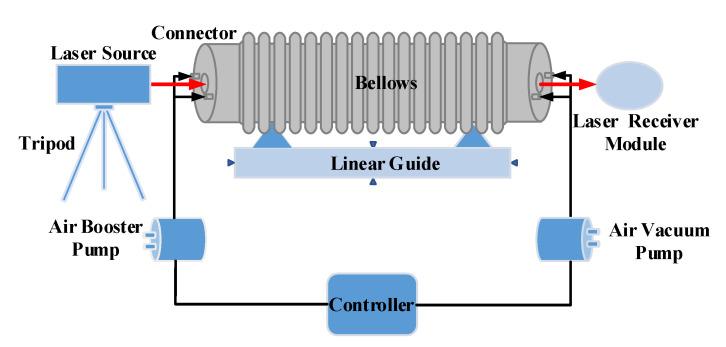
The schematic of the experimental scheme.

Figure 3 illustrates the schematic of the experimental scheme. Cylindrical connectors are installed both at the front and the end of the bellows to form a closed cavity. A circular window with a diameter of 35 mm is arranged at the center of the connectors. Two air inlet and exhaust ports are arranged symmetrically on both sides of the circular window where an optical glass is mounted. The bellows and connectors are horizontally fixed on a linear guide.

The laser beam, which is emitted by a laser source, injects in and out of the bellows through an optical glass and then comes to a laser receiver module. The laser source is installed on a tripod to ensure the stability. An air booster pump is connected to the inlet ports to inflate air into the bellows. An air vacuum pump is connected to the exhaust ports to pump the air out of the bellows. Accordingly, the axial temperature and pressure gradient can be controlled within the allowable error in the disturbed environment. The flow of the air inlet and exhaust is regulated by a controller by controlling the motor voltage of pumps, thus setting a suitable flow for different environmental interferences.

### 3.2. Parameter Optimization

#### 3.2.1. Inner Diameter of Bellows

When the external environmental interference is a uniform heat source, the axial temperature gradient in the bellows does not change significantly. Therefore, the parameter was optimized under a local heat source condition.

**Figure 4:**
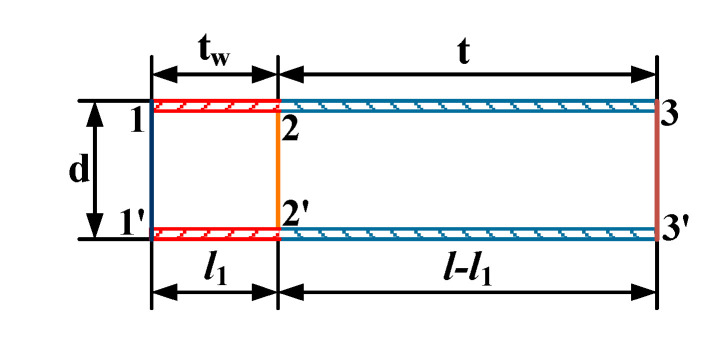
Computational model of fluid flow.

The computational model of fluid flow is shown in Figure 4. The length *l* of the bellows is 2 m. Air flows into the bellows from the cross section 1–1′ and flows out from the cross section 3–3′. Supposing that a local heat source with a length of *l*_1_ is applied to the bellow wall, the local temperature of the wall increases to *t_w_*. According to the balance equation for flow and heat transfer [24], the average airflow speed *u_m_* in the bellows can be calculated as follows:

(9) $$u_{m}=\frac{h\pi dl_{1}\left(t_{w}-t_{f1}\right)}{\frac{\pi d^{2}}{4}\rho_{1}c_{p}\left(t_{f2}-t_{f1}\right)}=\frac{4Nu\lambda_{f}l_{1}\left(t_{w}-t_{f1}\right)}{d^{2}\rho_{1}c_{p}\left(t_{f2}-t_{f1}\right)},$$

where *Nu* and *λ_f_* are the Nusselt number and thermal conductivity at temperature *t_f_*_1_, respectively; *d* is the inner diameter of the bellows; *t_f_*_2_ is the average temperature of the cross section 2–2′; and *c_p_* and *ρ*_1_ are the specific heat capacity of air and the air density at temperature *t_f_*_1_, respectively.

Similarly, the average temperature of the cross section 2–2′ can be calculated as

(10) $$\begin{array}{l} t_{f2}=\frac{4h’\left(l-l_{1}\right)\left(t_{f3}-t\right)}{d\rho_{2}c_{p}’u_{m}}+t_{f3}\\ \;\;\;\;\;=\frac{4Nu’\lambda_{f}’\left(l-l_{1}\right)\left(t_{f3}-t\right)}{d^{2}\rho_{2}c_{p}’u_{m}}+t_{f3} \end{array},$$

where *ρ*_2_ and *c_p_*′ are the air density and specific heat capacity of air at temperature *t_f_*_3_, respectively; and *Nu*′ and *λ_f_*′ are the Nusselt number and thermal conductivity at temperature *t_f_*_3_, respectively. On the basis of Equations (9) and (10), *u_m_* can be expressed as follows:

(11) $$u_{m}=\frac{4\left[Nu\lambda_{f}l_{1}\rho_{2}c_{p}’\left(t_{w}-t_{f1}\right)-Nu’\lambda_{f}’\rho_{1}c_{p}\left(l-l_{1}\right)\left(t_{f3}-t\right)\right]}{d^{2}\rho_{1}\rho_{2}c_{p}c_{p}’\left(t_{f3}-t_{f1}\right)},$$

The Reynolds number is a basic parameter that characterizes fluid flow. For the fluid flow in the bellows, it can be calculated as follows:

(12) $$Re=\frac{\rho u_{m}d}{\mu },$$

where *ρ* is the average air density *μ* is the air dynamic viscosity. When the Reynolds number is less than 2200, air flow in the bellows is laminar [25]. Therefore, the maximum average airflow speed in the bellows can be calculated as

(13) $${\left(u_{m}\right)}_{\max}=\frac{2200\mu }{\rho d},$$

Under the condition of laminar flow, *Nu* and *Nu*′ can be expressed as follows [26]:

(14) $$Nu={{Re}_{f}}^{1/3}{{Pr}_{f}}^{1/3}{\left(d/l_{1}\right)}^{1/3}{\left(\mu_{f}/\mu_{w}\right)}^{0.14},$$

(15) Nu’=(Ref’)1/3(Prf’)1/3[d/(l−l1)]1/3(μf’/μt)0.14,

where *Re_f_* and *Pr_f_* are the Reynolds and Prandtl numbers at temperature *t_f_*_1_, respectively; *μ_w_* is the air dynamic viscosity at temperature *t_w_*; *μ_f_* is the air dynamic viscosity at temperature *t_f_*_1_; *Re_f_*′ and *Prf*′ are the Reynolds and Prandtl numbers at temperature *t_f3_*, respectively; *μ_f_*′ is the air dynamic viscosity at temperature *t_f_*_3_; and *μ_t_* is the air dynamic viscosity at temperature *t*.

When the device is placed under normal temperature and pressure, the temperature of the air flowing into the bellows is at 20 °C. Therefore, the average temperature *t_f_*_1_ of the cross section 1–1′ and the wall temperature *t* of the bellows are both 20 °C. Based on Equation (7), the difference between the average temperature of cross sections 1-1′ and 3-3′ should not exceed 0.8 °C. Thus, the maximum average temperature *t_f_*_3_ of the cross section 3–3′ is 20.8 °C. On the basis of Equations (11), (14) and (15), when the length *l*_1_ of the local heat source is 300 mm, the average airflow speed *u_m_* in the bellows can be approximately calculated as follows:

(16) $$u_{m}\approx \frac{{\left[1.9\left(t_{w}-20\right)-5.0802\right]}^{3/2}}{1.25d^{2}}.$$

**Figure 5:**
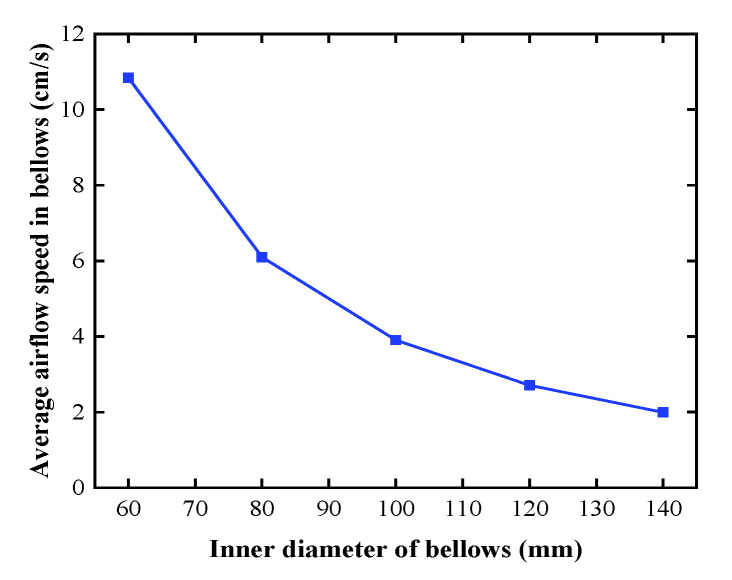
Average airflow speed in the bellows for different inner diameters.

Figure 5 shows the average airflow speed in the bellows for different inner diameters when a local heat source with a temperature of 23 °C is applied to the bellows wall in accordance with the combination of Equations (13) and (16). It can be seen that the larger the inner diameter is, the smaller the average airflow speed required to meet the requirements of the maximum allowable axial temperature gradient. Meanwhile, the impact of air disturbance on the laser beam can be reduced by increasing the inner diameter of the bellows. However, the cost of the air pumps and bellows increases with the diameter. When the inner diameter is greater than 100 mm, the average airflow speed decreases very slowly. Hence, an industrial bellows with an inner diameter of 102 mm is utilized in the experiment, which can meet the performance requirements.

#### 3.2.2. Determination of the Parameters and Positions of Optical Elements

**Figure 6:**
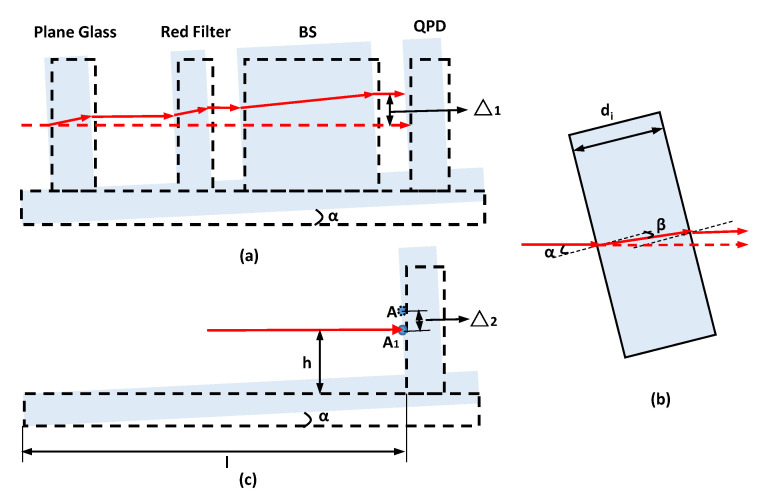
Model of Straightness error caused by angle offset, (**a**) is the principle of error Δ1, (**b**) is a local graph of (**a**), (**c**) is the principle of error Δ2.

Angle error and straightness error can be measured simultaneously for the proposed a 4-DOF measurement system. However, additional straightness error will occur due to the angle error of guide rail, which will reduce the measurement accuracy of straightness error. Hence, the coupling between the two errors needs to be reduced or eliminated by choosing appropriate parameters and positions of optical elements. As the QPD, BS, red filter, and flat glass are vertically mounted on the guide plate, an angle will occur between the laser beam and these optical elements in the case of a pitch or yaw of the guide plate (Figure 6a). When the inclination angle of the plate is α, the laser offset caused by refraction when the laser beam passes through an optical element is Δ_1_ (Figure 6b). The expression of straightness error caused by angle error is as follows:

(17) $$\Delta 1=\sum_{i=1}^{3}di(\tan\alpha -\tan\beta )\cos\alpha ,$$

where *d*_1_ is the thickness of plane glass, *d*_2_ is the thickness of red filter, *d*_3_ is the thickness of BS, α is the incident angle of laser beam, and β is the refraction angle of laser beam.

As shown in Figure 6c, the laser projects on the QPD at point A when there is no inclination angle for the flat plate, and the laser beam is incident at point A_1_ when the plate rotates an angle α. The distance between point A and point A_1_ is the straightness error Δ_2_, which can be defined as follows:

(18) $$\Delta 2=h-\frac{h}{\cos\alpha }+l\sin\alpha ,$$

where *h* is the height of the initial laser beam to the parallel plate, *l* is the distance from the QPD to the end of the plate.

Due to the opposite signs of △_1_ and △_2_, the two deviations can cancel each other by setting appropriate values of *d*_1_*, d*_2_*, d*_3_*, h,* and *l*. Since *d*_2_*, d*_3,_ and *h* are not suitable to be changed, the two kinds of errors can be cancelled out in the range of 0 to 100 angular seconds by changing *d*_1_ and *l.* Accordingly, when *l* is 33 mm and *d*_1_ is 10 mm, the two errors can be completely offset. Hence, the coupling between the two errors can be totally eliminated.

#### 3.2.3. Control Mode for the Pumps

As an important basic parameter for incompressible flow, Mach number is defined as the ratio of the speed of the fluid (*u*) to the speed of sound (*a*) in the fluid under the conditions of flow [27].

(19) $$N_{Ma}\equiv \frac{u}{a},$$

According to Equation (13), when the inner diameter of the bellows is 102 mm, the maximum average airflow speed in the bellows is 33 cm/s. According to Equation (19), the Mach number is far less than 0.3. At a low Mach number, the process conforms essentially to the usual Bernoulli relation for incompressible flow [28]. Therefore, as shown in Figure 5, the average pressure difference ∆*p* between the two cross sections can be calculated by using Bernoulli’s equation as follows:

(20) $$\begin{array}{l} \Delta p=\rho_{m}\left(U_{1}+z_{1}g+\frac{1}{2}{u_{1}}^{2}+W_{e}+Q_{e}\right)-\rho_{m}\left(U_{2}+z_{2}g+\frac{1}{2}{u_{2}}^{2}\right)\\ \;\;\;\;\;\;=\frac{\rho_{1}+\rho_{2}}{2}\left(W_{e}+Q_{e}+\Delta U+\Delta zg+\frac{1}{2}\Delta u^{2}\right) \end{array},$$

where ∆*U* is the internal energy difference between the two cross sections, ∆*z* is the distance difference from the center of the two cross sections to the reference level, ∆*u* is the average airflow speed difference between the two cross sections, *W_e_* is the effective work of external forces, and *Q_e_* is the external heat energy.

Given that no effective work of external force is generated during the fluid flow and the bellows is placed horizontally,

(21) $$W_{e}=0,\;z_{1}=z_{2},$$

Considering that the internal energy of the actual fluid flow is quite complicated, the following approximation is customarily adopted [29]:

(22) $$\Delta U=C_{V}\Delta T,$$

where *C_v_* is the specific heat at constant volume.

Equation (20) can thus be derived as follows:

(23) $$\begin{array}{l} \Delta p=\frac{\rho_{1}+\rho_{2}}{2}\left(Q_{e}+\Delta U+\frac{1}{2}\Delta u^{2}\right)\\ \;\;\;\;\;=\frac{\rho_{1}+\rho_{2}}{2}\left(Q_{e}+C_{V}\Delta T+\frac{1}{2}\Delta u^{2}\right) \end{array},$$

**Figure 7:**
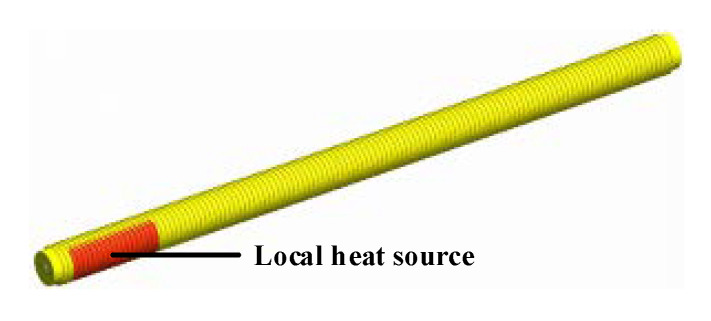
Simplified solid model of the bellows.

By controlling the pumps located at both ends of the bellows to be open or closed, four control modes can be realized to obtain the optimal parameters in the bellows, including without air inlet and exhaust, only air inlet, only air exhaust, and with air inlet and exhaust. ANSYS CFX software is employed for simulation to choose an appropriate mode and ensure that the air temperature and pressure in the bellows are stable. The simulation model and other parameters of the four control modes are the same. In the last mode, the speed of the air inlet and exhaust can be set as greater than, less than, or equal to the exhaust speed. Under the same conditions, from Equation (23), the average pressure difference between the two cross sections is the smallest only if the speed of the air inlet and exhaust are equal (∆*u* = 0). Thus, the two speeds are set to be the same in the simulation. Figure 7 shows the simplified solid model of the bellows with length, inner diameter, and wall thickness of 2 m, 102 mm and 6 mm, respectively. The turbulent model is the standard k–ε model, and the condition of the bellows wall is a standard wall surface function. The material of the fluid is air, with temperature and pressure at 20 °C and 1 bar, respectively. The temperature is unstable throughout the entire propagation trajectory of the optical path only if a local heat source is arranged near the air inlet ports. Under this condition, the temperature interference is high, and the parameters in the bellows are difficult to control. Therefore, the heat source is applied to the local semicircle wall near the air inlet ports to select the optimum control mode. The temperature is set to 23 °C, and the length is 300 mm.

**Figure 8:**
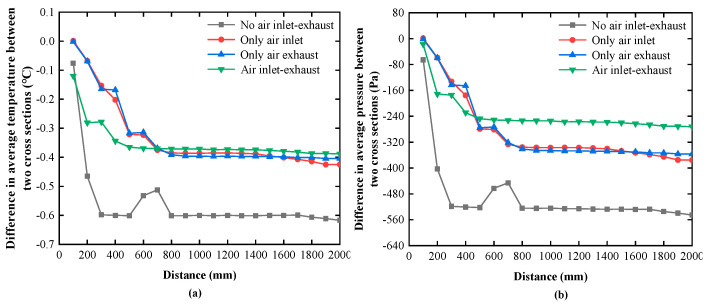
Difference in average temperature (**a**) and average pressure (**b**) between the two cross sections.

The average temperature difference between the two cross sections, as shown in Figure 8a, can be directly analyzed through the simulation tool. However, the average pressure difference between the two cross sections must be calculated using Equation (23), as shown in Figure 8b. The circumstance with the air inlet and exhaust stands out from the three other modes because either the average temperature or the average pressure difference between the two cross sections is small. The simulation results are tabulated in Table 1. In the desired mode, the maximum axial temperature and pressure gradients are 0.15 °C/m and 136 Pa/m, respectively, both of which meet the design requirements. Hence, the mode with air inlet and exhaust is utilized in this study, and the air inlet speed is set to be equal to the exhaust speed in experiments.

## 4. Experimental Setup and Results

**Figure 9:**
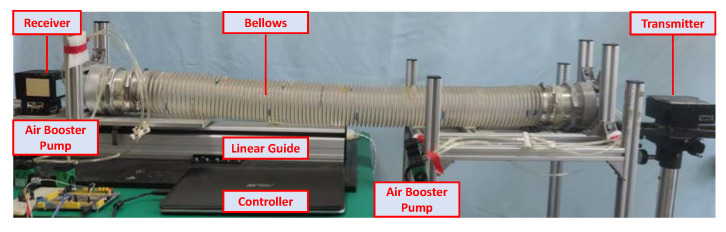
Photo of the experimental setup.

An experimental setup was constructed on a 4-DOF measurement system to verify the feasibility of the proposed method, as shown in Figure 9. The receiver and transmitter of the 4-DOF measurement system are located at both end of the bellows. The bellows is a type of scalable, high-temperature and wear-resistant circular pipe (MP06). The air booster pump (GEBS12120P802C) and air vacuum pump (GEBS1272805C) are both micro pumps. The flow and airflow speed can be controlled through the motor voltage of pumps. The controller is a development board based on ARM. The laser beam drift is measured by the laser receiver module, which is placed at a fixed position of 2 m away from the laser source.

**Figure 10:**
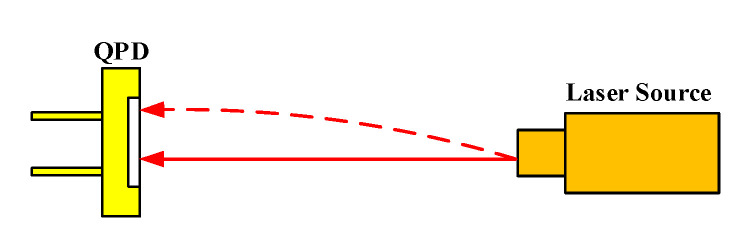
Optical configuration of the laser receiver module.

The optical configuration of the laser receiver module is shown in Figure 10. When the parallel laser beam is launched from the laser source incident upon the QPD, an adjusting mechanism is used to tune the lights and focus them to the center of the QPD. When the laser beam drift occurs, the light spot on the surface of the QPD shifts, and its displacement can be detected by the QPD.

**Figure 11:**
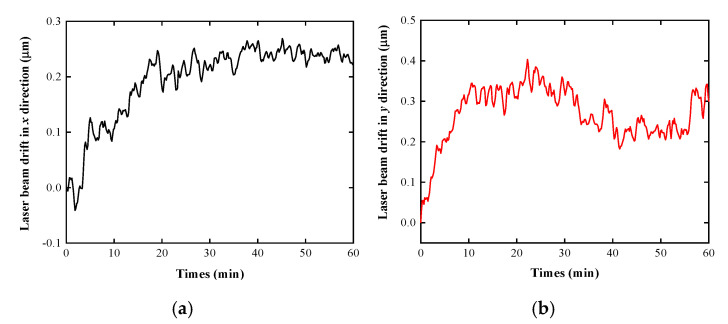
Experimental results of the laser beam drift in (**a**) *x* and (**b**) *y* directions under normal ambient condition without external temperature interference.

**Figure 12:**
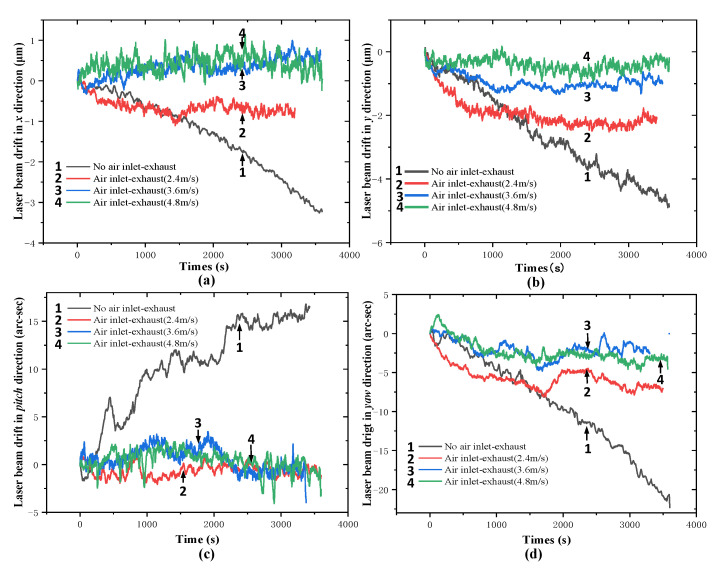
Experimental results of the laser beam drift in (**a**) *x*, (**b**) *y*, (**c**) *pitch,* and (**d**) *yaw* directions under normal ambient condition with a local heat source.

Under normal ambient condition, the light spot is adjusted to the center of the QPD, and the values of the light spot on the QPD are recorded every 1 s. Figure 11 shows the results of the laser beam drifts in the x and y directions without external temperature interference. Figure 12 reveals the experimental results of the laser beam drift in the x, y, pitch and yaw directions when a local heat source is simulated by an incandescent lamp (60 W) under the normal ambient condition after trying to adjust the flow of air inlet and exhaust.

Figure 11 indicates that the laser beam drift without external temperature interference in the *x* direction is 0.4 μm and that in the *y* direction is 0.5 μm. As shown in Figure 12, the laser beam drift is gradually reduced with the increase of the pump’s current velocity. When the pump’s current velocity is adjusted to 4.8 m/s, the position stability of the laser beam spot is significantly improved. The laser beam drift in the *x* direction without air inlet–exhaust is 3.4 μm while that with air inlet–exhaust (4.8 m/s) is only 0.7 μm. Hence, in the mode of air inlet and exhaust, the position stability of the laser beam spot is improved by 79.41%. Similarly, in the *y* direction, the laser beam drift is 5.0 μm without air inlet–exhaust while that with air inlet and exhaust is 0.5 µm. The position stability of the laser beam spot with the air inlet and exhaust is improved by 90%. The position stability of the laser beam spot with air inlet and exhaust is evidently improved in the pitch and yaw direction. The experimental results are summarized in Table 2.

The experimental results indicate that the laser beam drift in the *x* and *y* direction is 79.41% and 90%, which meets the design requirements. As can be seen that the proposed method can reduce the laser beam drift in long-distance measurement under the condition of local temperature interference. Hence, the method can be employed to improve the position stability of the laser beam spot in the laser measurement system.

## 5. Conclusions and Discussion

This study proposes a method of stabilizing the air refractive index to reduce the influence of laser beam drift on the detection accuracy of the laser measurement system. A technical scheme with the utilization of bellows and air pump was implemented to create a stable propagation environment for the laser optical path. The appropriate inner diameter of 102 mm for the bellows was chosen via theoretical analysis. The control mode of the pumps was optimized by fluid simulation and then the mode of air inlet and exhaust was utilized. A series of experiments were conducted to verify the feasibility of the proposed method. Under normal ambient conditions with local temperature interference, the position stability of the laser beam spot by air inlet and exhaust could be improved by 79.41% and 90% in the x and y direction. The position stability of the laser beam spot with air inlet and exhaust is evidently improved in the pitch and yaw direction. The better suppression effect of laser beam drift can be realized when the temperature of heat source is higher. Experimental results indicate that the proposed scheme is a feasible method that can reduce the laser beam drift caused by the unstable air refractive index. It can be applied to improve the detection accuracy of the 4-DOF measurement system. Further study will be focused on the selection of appropriate air flow according to the temperature of the heat source, thus avoiding the error caused by the interference of high flow rate.

## Figures and Tables

**Table 1:** Simulation results.

Simulation Results	Modes			
	No Air Inlet-Exhaust	Only Air Inlet	Only Air Exhaust	Air Inlet-Exhaust
Axial temperature gradient (°C/m)	0.30	0.20	0.20	0.15
Axial pressure gradient (Pa/m)	272	187	178	136

**Table 2:** Experimental results of the laser beam drifts in x and y directions.

Experimental Conditions		Laser Beam Drifts	
		*x* Direction (μm)	*y* Direction (μm)
No Interference		0.3	0.4
Interference (A local heat source)	No air inlet-exhaust	3.4	5.0
	Air inlet-exhaust (2.4 m/s)	1.2	2.5
	Air inlet-exhaust (3.6 m/s)	0.9	1.4
	Air inlet-exhaust (4.8 m/s)	0.7	0.5
